# Supplementary figures and images for: Do restoration strategies in mangroves recover microbial diversity? A case study in the Yucatan peninsula
Source: PLoS One. 2024 Aug 16;19(8):e0307929. doi: 10.1371/journal.pone.0307929 (PMC11329136; doi:10.1371/journal.pone.0307929)

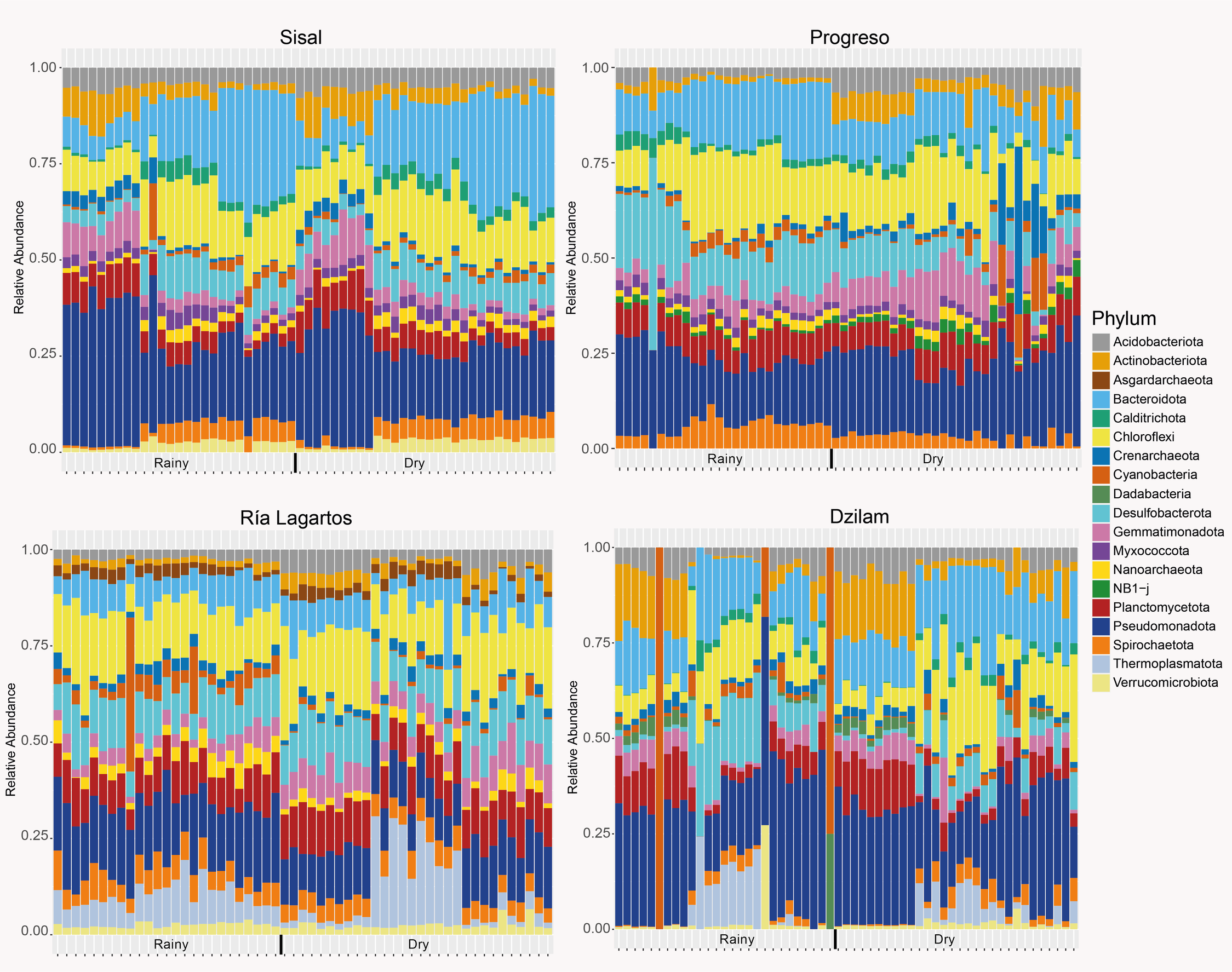

Supplement: S1 Fig — Relative abundance (y-axis). Bottom labels are the samples and their corresponding climatic season. (TIF) [file pone.0307929.s001.tif]

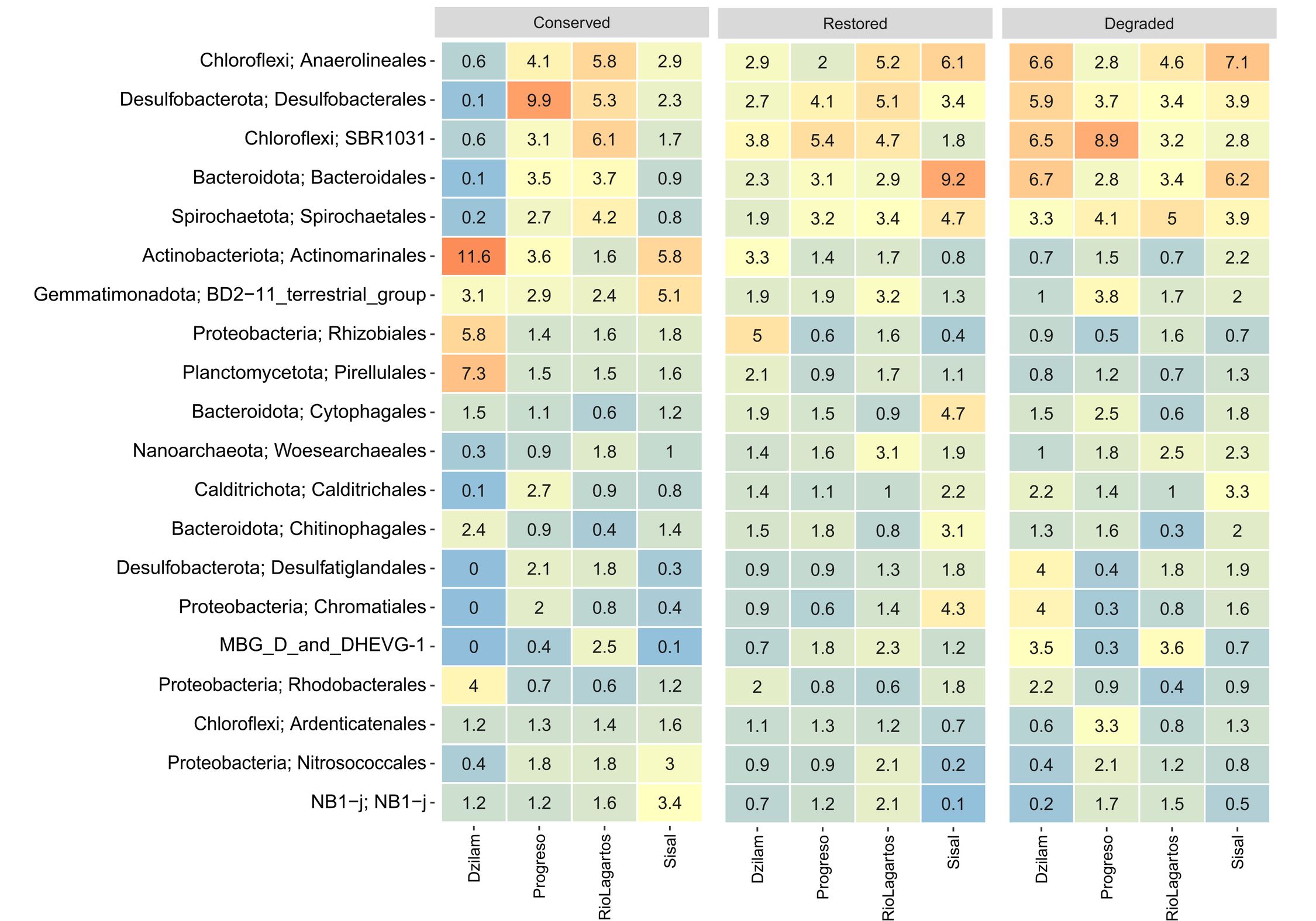

Supplement: S2 Fig — Relative abundances arranged by mangrove conservation condition (horizontal axis upper label) and the four study sites (horizontal axis lower label). (TIF) [file pone.0307929.s002.tif]

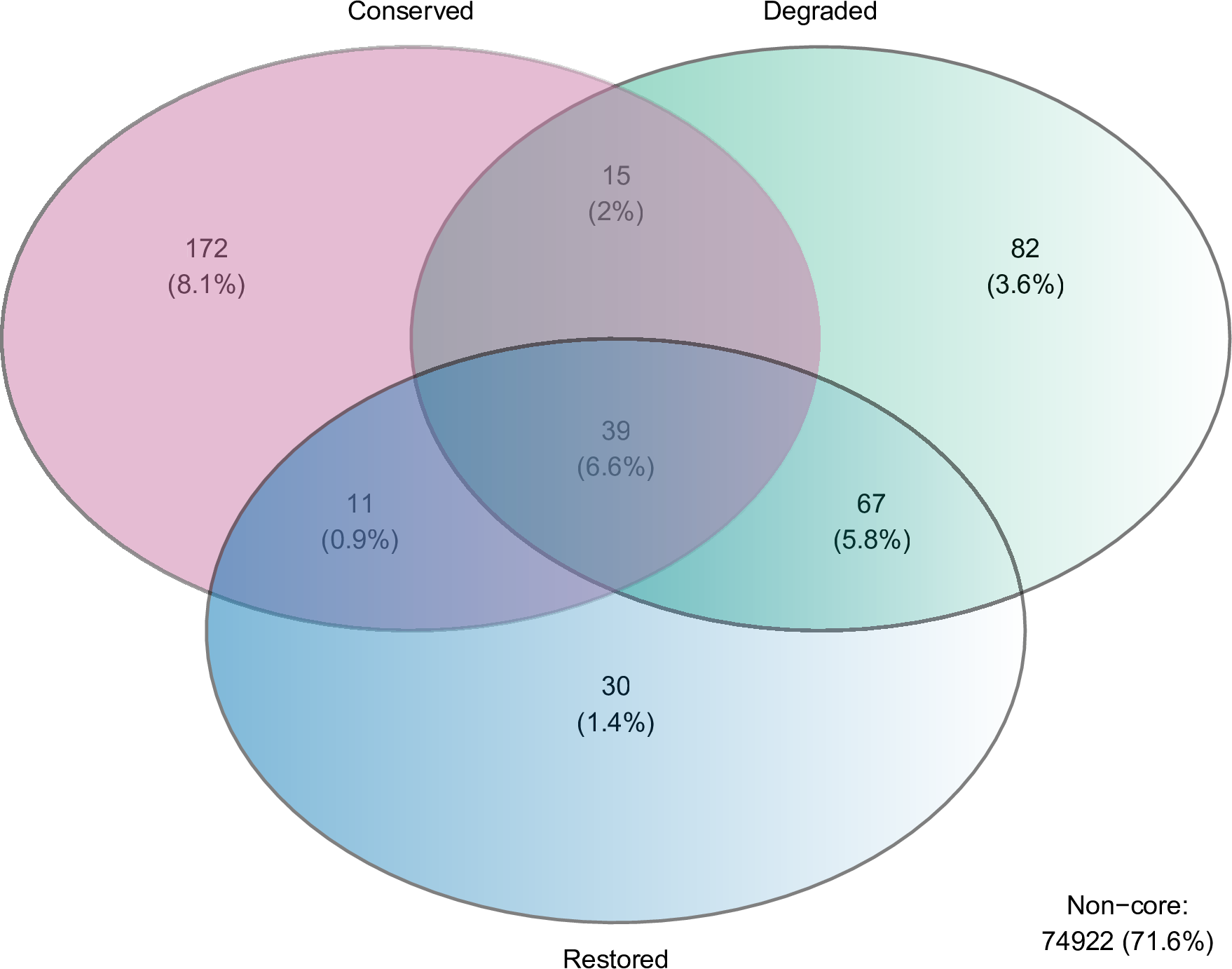

Supplement: S3 Fig — Shows the core microbiome (50% prevalence in the group pictured, reads with abundances above 0.1%) along the conservation status. The values inside the ellipses are the number of core ASVs and in parentheses the average relative abundance. (TIF) [file pone.0307929.s003.tif]

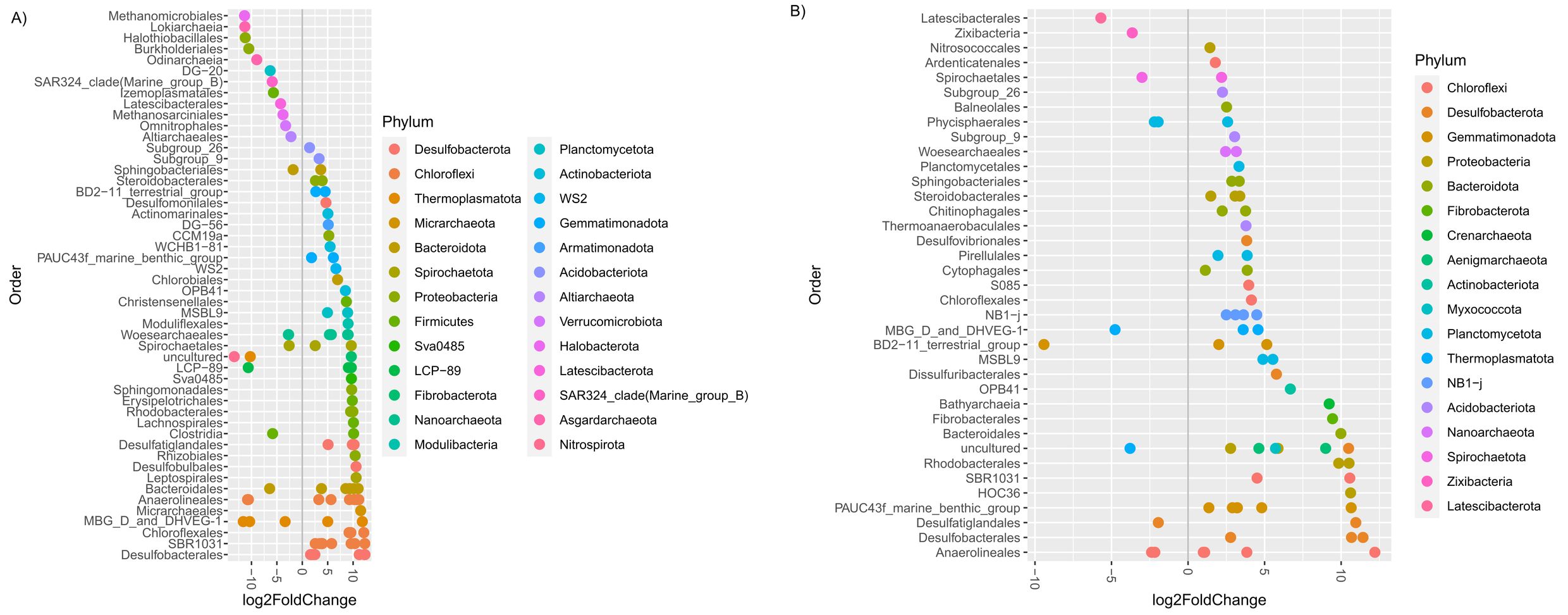

Supplement: S4 Fig — Log2 fold change comparing differentially abundant taxa (positive values are ASVs that have significantly higher abundance than degraded samples, while negative values are ASVs that are significantly less abundant than degraded samples). (A) Compares Conserved vs Degraded. (B) Compares Restored vs Degraded. (TIF) [file pone.0307929.s004.tif]

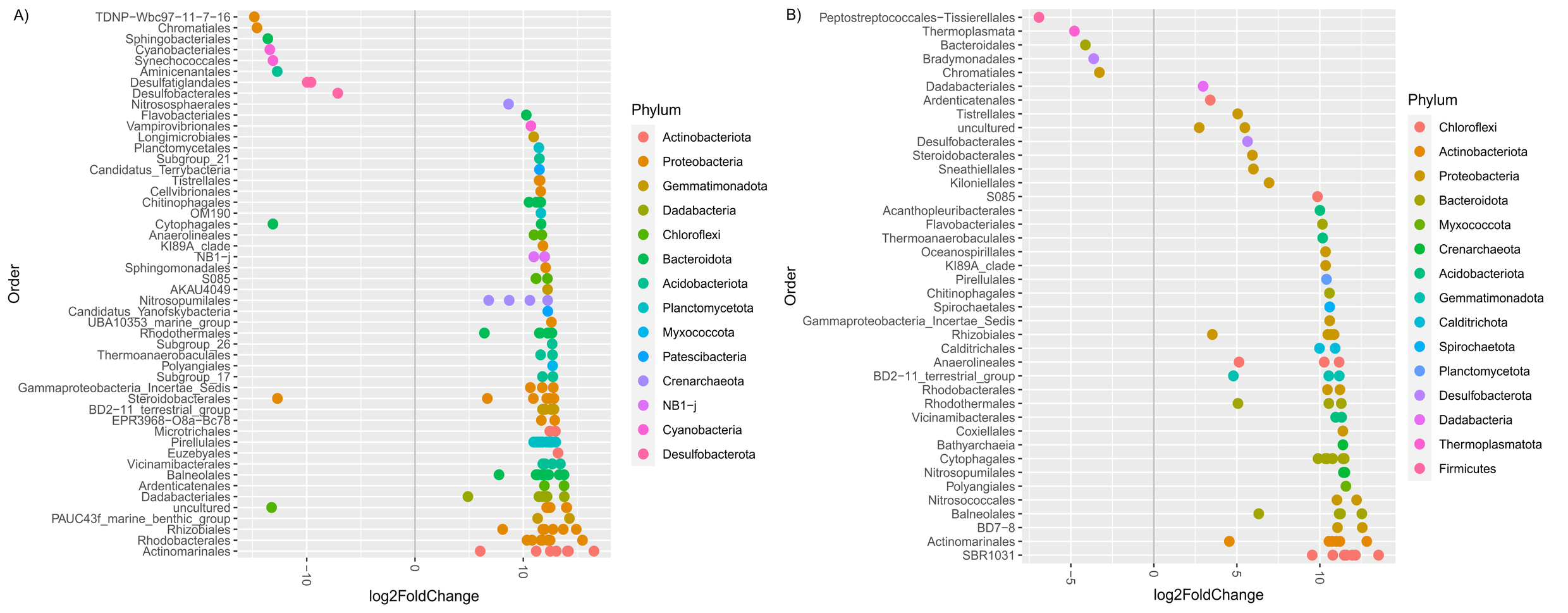

Supplement: S5 Fig — Log2 fold change comparing differentially abundant taxa (positive values are ASVs that have significantly higher abundance than degraded samples, while negative values are ASVs significantly less abundant than degraded samples). (A) Compares Conserved vs Degraded. (B) Compares Restored vs Degraded. (TIF) [file pone.0307929.s005.tif]

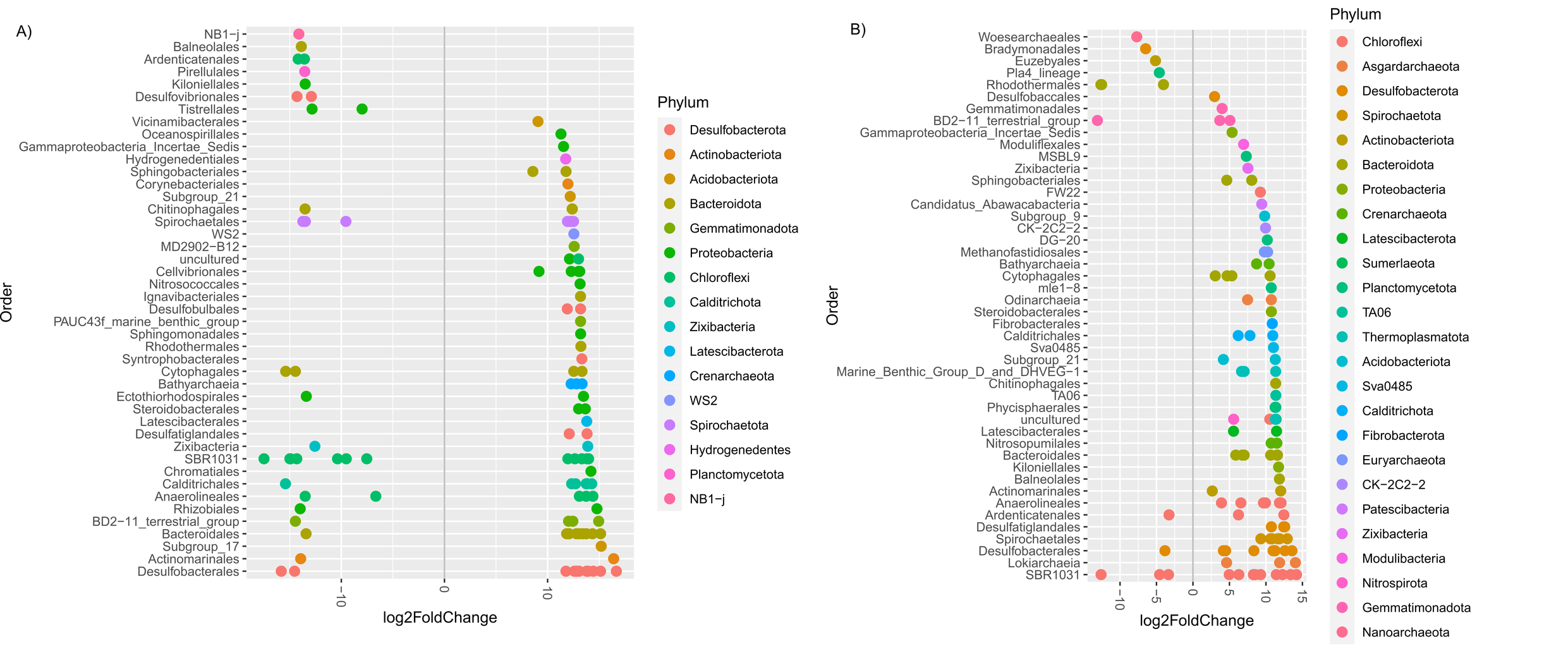

Supplement: S6 Fig — Log2 fold change comparing differentially abundant taxa (positive values are ASVs that have significantly higher abundance than degraded samples, while negative values are ASVs significantly less abundant than degraded samples). (A) Compares Conserved vs Degraded. (B) Compares Restored vs Degraded. (TIF) [file pone.0307929.s006.tif]

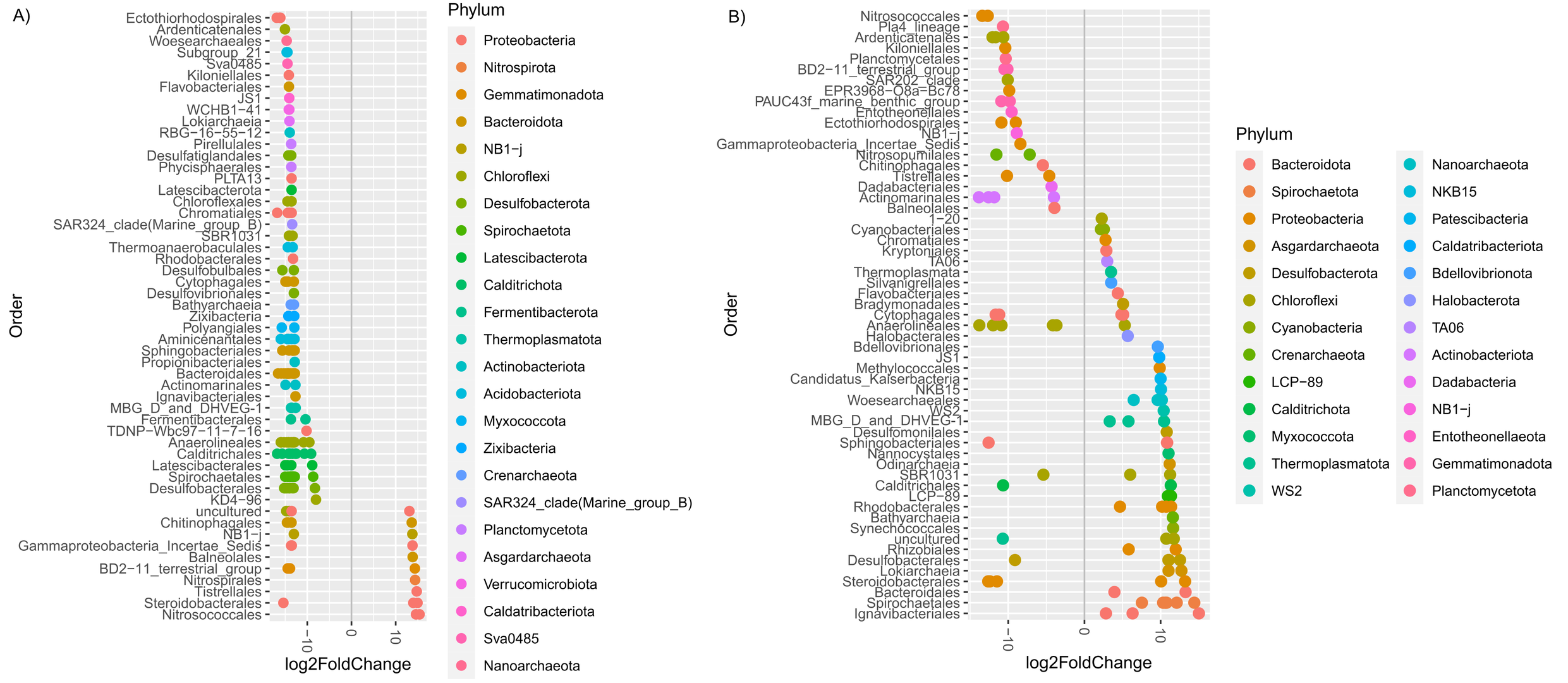

Supplement: S7 Fig — Log2 fold change comparing differentially abundant taxa (positive values are ASVs that have significantly higher abundance than degraded samples, while negative values are ASVs significantly less abundant than degraded samples). (A) Compares Conserved vs Degraded. (B) Compares Restored vs Degraded. (TIF) [file pone.0307929.s007.tif]
