# Supplementary material for: Do restoration strategies in mangroves recover microbial diversity? A case study in the Yucatan peninsula
Source: PLoS One. 2024 Aug 16;19(8):e0307929. doi: 10.1371/journal.pone.0307929 (PMC11329136; doi:10.1371/journal.pone.0307929)
Supplement: S2 Table — (PDF) [file pone.0307929.s009.pdf]

Site	Pair of conditions	Sum of squares	R^2	F	p-value
**R. Lagartos**	**Conserved vs Degraded**	0.005304	0.20194	7.8444	< 0.001
	**Conserved vs Restored**	0.0031436	0.11089	3.9912	< 0.001
	**Degraded vs Restored**	0.0038176	0.13498	5.1492	< 0.001
**Dzilam**	**Conserved vs Degraded**	0.009470	0.3988	21.891	< 0.001
	**Conserved vs Restored**	0.0042526	0.19104	7.7934	< 0.001
	**Degraded vs Restored**	0.0040663	0.16279	6.2221	< 0.001
**Progreso**	**Conserved vs Degraded**	0.0068949	0.42544	25.175	< 0.001
	**Conserved vs Restored**	0.0041388	0.26649	11.626	< 0.001
	**Degraded vs Restored**	0.0027378	0.17022	6.9748	< 0.001
**Sisal**	**Conserved vs Degraded**	0.0061954	0.51997	36.83	< 0.001
	**Conserved vs Restored**	0.0077090	0.60157	51.335	< 0.001
	**Degraded vs Restored**	0.0025862	0.38672	21.439	< 0.001
