# Supplementary material for: Do restoration strategies in mangroves recover microbial diversity? A case study in the Yucatan peninsula
Source: PLoS One. 2024 Aug 16;19(8):e0307929. doi: 10.1371/journal.pone.0307929 (PMC11329136; doi:10.1371/journal.pone.0307929)
Supplement: S3 Table — Shows the values of the multiple physical-chemical parameters that cause the divergence between the conservation status. (PDF) [file pone.0307929.s010.pdf]

Site	Variable	Sum of squares	R^2	F	p-value
**R. Lagartos**	**Corg**	0.8038	0.06251	3.8810	< 0.001
	**Temperature**	0.6491	0.05048	3.1341	< 0.001
	**Redox**	1.1695	0.09095	5.6470	< 0.001
	**Salinity**	0.6000	0.04666	2.8970	0.003
	**pH**	0.3171	0.02466	1.5311	0.076
	**Residual**	9.3198	0.72475		
	**Total**	**12.8593**	**1.00000**		
**Dzilam**	**Corg**	1.3630	0.06964	3.9777	< 0.001
	**Temperature**	0.4822	0.02464	1.4072	0.154
	**Redox**	0.6964	0.03558	2.0322	0.039
	**Salinity**	0.6757	0.03452	1.9718	0.048
	**pH**	0.5910	0.03020	1.7246	0.065
	**Residual**	15.7631	0.80541		
	**Total**	**19.5714**	**1.00000**		
**Progreso**	**Corg**	3.3948	0.19091	14.4064	< 0.001
	**Temperature**	1.2893	0.07250	5.4713	< 0.001
	**Redox**	0.9304	0.05232	3.9483	0.002
	**Salinity**	0.5396	0.03034	2.2899	0.014
	**pH**	0.5529	0.03109	2.3462	0.019
	**Residual**	11.0754	0.62283		
	**Total**	**17.7824**	**1.00000**		
**Sisal**	**Corg**	5.2076	0.34300	35.9108	< 0.001
	**Temperature**	0.4722	0.03110	3.2561	0.030
	**Redox**	1.4168	0.09332	9.7701	< 0.001
	**Salinity**	0.4335	0.02855	2.9892	0.028
	**pH**	0.6918	0.04556	4.7703	0.006
	**Residual**	6.9608	0.45847		
	**Total**	**15.1827**	**1.00000**		
