# Supplementary material for: Do restoration strategies in mangroves recover microbial diversity? A case study in the Yucatan peninsula
Source: PLoS One. 2024 Aug 16;19(8):e0307929. doi: 10.1371/journal.pone.0307929 (PMC11329136; doi:10.1371/journal.pone.0307929)
Supplement: Attachment — Submitted filename: Response_to_Reviewers.pdf [file pone.0307929.s021.pdf]

Jiang-Shiou Hwang, Ph.D.

Academic Editor

PLOS ONE

Dear Dr. Hwang,

We hereby include the response to the observations made during the revision of the following article **"Do restoration strategies in mangroves recover microbial diversity? A case study in the Yucatan peninsula"**, an original research work by Daniel Esguerra, Arit De León, Claudia Teutli, Alejandra Prieto-Davó, José Q García-Maldonado, Jorge Herrera-Silveira and Luisa I Falcón.

We appreciate your remarks, and in attendance to the journal requirements, the manuscript has been modified to assure it complies with the format and style guidelines. Particularly, authors' affiliations were corrected, and some references cites were also set accordingly. Also, in the methods section "Study site and sample collection" (see lines 142-144), we state the permit number emitted by the national environmental agency (Secretaría de Medio Ambiente y Recursos Naturales) to collect the samples of the study. To attend to the code sharing guidelines and policies, in the corresponding methods sections, a release from the *GitHub* public repository from author DER is referenced with a DOI link (https://doi.org/10.5281/zenodo.11122245) to access the code to analyze the data and recreate the map of the study area (Fig 1), the folder is public and available without restrictions.

Regarding the acknowledgements section, details of founding instances were removed, and we add the state that declares founders had no role in the study design, data collection and analysis, decision to publish, or manuscript preparation.

To enable the data availability for the reviewing process we generated a reviewer link (https://dataview.ncbi.nlm.nih.gov/object/PRJNA1039151?reviewer=5pp16fdm50h2ksup5mv8mqa5qq) to the sequences that are already part of the *NCBI* repository. This link will give access to reviewers and editors to the *Bioproject* and associated SRA metadata with the sequences. Complete free access to the entire data upon publication is already set for the corresponding *Bioproject* in *NCBI*.

Regarding the copyrights concerns on the study sites map (Fig 1), we replaced the figure and stated that its construction was made from vectorial data that are freely available for academic use and can be published under an open license CC BY. The references of the vectorial data sources were included in the caption, as was the link to the code that reproduces the figure.

We appreciate your time and attention, and we look forward to receiving your comments.

Yours sincerely

Daniel Esguerra and Luisa Falcon

Attending the reviewers' comments to the authors, the following actions were taken:

Reviewer 1 suggestions:

1. In materials and methods, it is recommended to define protected, degraded, and restored mangroves. It also points out the habitat differences between protected, degraded, and restored mangroves.
2. It is necessary to indicate whether the tides are different at the four sampling sites.

We appreciate your comments, the previous version was lacking specific descriptions of the sampling sites, and the key characteristics that could affect the microbial composition were not stated explicitly. To clarify these points, we added details that describe the sampling sites on lines 101-114, along with the important monitoring reference from "Laboratorio de Produccion Primaria – CINVESTAV (Herrera-Silveira et al., 2018)". We included the raw physicochemical information associated with the sediment samples (S1 Table), and critical characteristics of the sampling sites including the mean flooding level, where is evident that there are no differences in tides between the sites (Table 1). Average values of the physicochemical data were also calculated to better understanding of the parameters influencing sampling sites in each conservation status (Table 2). Dominant mangrove species was not the same among locations and conservation status, along with the tables and methods description, the relevance of each species is stated throughout the new version of the text.

Reviewer 2 suggestions:

1. In this study, results show that microbial diversity in sediments can be used as an index to indicate the environmental health in mangroves. I appreciate the effort to carry out studies that denote new ecological bioindicators, since it is a way to visualize the “ecological future, sustainably through the biota”. However, in my opinion, the purpose and the relevance of this specific study is still unclear. I hope that my comments are useful for the improvement of the article.

We appreciate the comments, and addressed the observations concerning the purpose, novelty and significance of the study; please refer to the introduction on lines 72-77 and 83-96, where we state the relevance of identifying potential indicator consortia as environmental assessors of the ecosystem. Furthermore, in the discussion section “Potential of microbes to monitor restoration”, we discuss the relevance of specific taxa in ecosystem functionality (see lines 426-454). Describing this range of microbes and its associations with specific conservation conditions highlights the value of a microbial baseline in hydrological restoration scenarios.

To attend major concerns, the following actions were taken:

1. To address the lack of clarity in our previous version we rewrote and restructured the manuscript. As stated in the last suggestion response we clarified the purpose, novelty, and significance of the study (see lines 72-77; 83-96). We also incorporated updated information and recent citations that help to understand the current state of knowledge of the subject. Throughout the manuscript, special attention was taken to the potential drivers of variation of the microbial composition, mangrove species, physicochemical variables and hydrological traits.

2. To address language adjustment and avoidance of redundancy, we asked a native professional to proofread the entire manuscript; we changed the wording, taking care to avoid repetitive sentences.

3. We appreciate your concern; we made several changes mainly to the introduction and discussion sections, including sentences that contextualize the importance within the field, for example, in lines 58-61, “Furthermore, there is little understanding of microorganisms' response and specific role in maintaining conserved mangroves, supporting restoration processes, and enduring degradation since most studies aim to understand local particular processes that influence microbial patterns.” In lines 72-91 “The mangroves in the YP have been degraded because of land use changes, infrastructure, urban expansion, pollution, and extreme climatic events. Many of these impacts have been identified and successful rehabilitation programs have been designed for this region. Although knowledge of microbial component variation associated to different conditions of mangrove forests in the YP has only started to be incorporated in local projects. Work in the Red Sea and in Colombia suggests differences in microbial composition in mangroves with higher anthropic impact than in pristine ecosystems, generally showing higher abundance of microbial taxa that potentially respond to disturbance in degraded areas. Further, studies in China highlight that mangrove restoration stimulates microbial diversity recovery in sediments, nutrient

availability and extracellular polymeric substance production. A recent study of the sediment microbial component in Celestun (northwest YP), reported that the red mangrove, *Rhizophora mangle,* in different ecological types had a more cosmopolitan and heterogenous composition than the less abundant black mangrove, *Avicennia germinans*, that hosts a specific microbiome, suggesting the relevance of incorporating microbial ecology studies to better define restoration strategies of mangrove forests. Hence, the aim of this study was to make a description of the composition of bacteria and archaea from mangrove sediments along the northern YP coast incorporating mangroves in different status: conserved, degraded and restored."

4. We appreciate your suggestion. We changed the description of the sampling sites and their characteristics to the methodology section and synthesized site characteristics in Tables 1 and 2.

5. Thanks for your accurate comment; we modified the abstract including specific relative abundance values of major phyla found, we wrote on lines 28-31 "Results showed that although each sampling site had a differentiated microbial composition, the taxa were mainly from Pseudomonadota (13.2 – 23.6%), Desulfobacterota (7.6 - 8.3%), and Chloroflexi (9 – 15.7%) phyla, these were similar between rainy and dry seasons". We also incorporated the significance values for the alpha diversity conservation status comparisons (see lines 32-34) and stated that some ASVs had significant abundance differences when degraded and conserved samples were compared (see lines 36-38). In this later case, significance values were not explicit since several ASVs are representing the differences within the mentioned taxonomical groups; significance values are referenced in the results section as supplementary tables of the differential abundance analysis (S5 Table – S13 Table).

6. We added specificity to the results stated in the abstract, as exemplified on the previous point to avoid ambiguity.

7. Thank you for the accurate observation, we modified the introduction and made sure novelty and significance were clearly stated, as mentioned in the response to major concern number 3.

8. Fig 1 was reconstructed, and areas were adjusted correctly, making sure that different vectorial data were at the same scale (1:50000), and the demarcations of coordinates in specific sites were added. The figure's general quality was enhanced, including a layer that represents the mangrove vegetation coverage.

9. Thanks for the comment. We replaced lines 90-118 and introduced Tables 1 and 2, which mention each sampling site and its characteristics.

10. Thanks for the accurate observation. Considering the more detailed site description and background research from the area, we discussed the possible explanations for the diversity values found in Sisal. Specifically, we highlight that in the degraded plots of Sisal the alpha

diversity result was exceptional (see lines 207-209), and we discussed how hydrologic flux linked to seawater and other characteristics may explain this result (see lines 371-384).

11. 12. and 13. Values of the abundances of the reported taxonomical groups were included. Taxa reported with higher abundance in the results section “Sediment microbial diversity and composition” were discussed in comparison to reference conditions (See lines 214-226). The significance values of the taxa that represented differences in contrasting conservation status are referenced in the section “Microbial differential abundance”, where more specific results of these analysis were included in supplementary tables (S5 Table – S13 Table).

14. Results section was reconstructed, including its subtitles. We gave the specific abundance values and included in text and supplementary information that show the significance level of the results, particularly in the comparative context of the different conservation status.

15. The discussion section was rewritten and restructured. The new version highlights the ecological relevance of our results and the variables that explain them; for example, in lines 442-457 we discuss the importance of the effect of the vegetation on microbial composition, as well as the effect of hydrological restoration in ecosystemic succession dynamics. The complete discussion section also gives details of the contexts where our findings can impact the most.
